# Novel Porous Phosphorus–Calcium–Magnesium Coatings on Titanium with Copper or Zinc Obtained by DC Plasma Electrolytic Oxidation: Fabrication and Characterization

**DOI:** 10.3390/ma11091680

**Published:** 2018-09-11

**Authors:** Krzysztof Rokosz, Tadeusz Hryniewicz, Sofia Gaiaschi, Patrick Chapon, Steinar Raaen, Dalibor Matýsek, Łukasz Dudek, Kornel Pietrzak

**Affiliations:** 1 Division of BioEngineering and Surface Electrochemistry, Department of Engineering and Informatics Systems, Koszalin University of Technology, Racławicka 15-17, PL 75-620 Koszalin, Poland; Tadeusz.Hryniewicz@tu.koszalin.pl (T.H.); lukasz.dudek@tu.koszalin.pl (Ł.D.); kornel.pietrzak@s.tu.koszalin.pl (K.P.); 2 HORIBA FRANCE S.A.S., Avenue de la Vauve, Passage Jobin Yvon, 91120 Palaiseau, France; sofia.gaiaschi@horiba.com (S.G.); patrick.chapon@horiba.com (P.C.); 3 Department of Physics, Norwegian University of Science and Technology (NTNU), Realfagbygget E3-124 Høgskoleringen 5, 7491 NO Trondheim, Norway; steinar.raaen@ntnu.no; 4 Institute of Geological Engineering, Faculty of Mining and Geology, VŠB—Technical University of Ostrava, 708 33 Ostrava, Czech Republic; dalibor.matysek@vsb.cz

**Keywords:** micro arc oxidation, plasma electrolytic oxidation, DC PEO, DC MAO, titanium, calcium nitrate tetrahydrate, magnesium nitrate hexahydrate, copper(II) nitrate trihydrate, 85% phosphoric acid

## Abstract

In this paper, the characteristics of new porous coatings fabricated at three voltages in electrolytes based on H_3_PO_4_ with calcium nitrate tetrahydrate, magnesium nitrate hexahydrate, and copper(II) nitrate trihydrate are presented. The SEM, energy dispersive spectroscopy (EDS), glow discharge optical emission spectroscopy (GDOES), X-ray photoelectron spectroscopy (XPS), and XRD techniques for coating identification were used. It was found that the higher the plasma electrolytic oxidation (PEO) (micro arc oxidation (MAO)) voltage, the thicker the porous coating with higher amounts of built-in elements coming from the electrolyte and more amorphous phase with signals from crystalline Ca(H_2_PO_4_)_2_∙H_2_O and/or Ti(HPO_4_)_2_∙H_2_O. Additionally, the external parts of the obtained porous coatings formed on titanium consisted mainly of Ti^4+^, Ca^2+^, Mg^2+^ and PO_4_^3−^, HPO_4_^2−^, H_2_PO_4_^−^, P_2_O_7_^4−^ as well as Zn^2+^ or copper Cu^+^/Cu^2+^. The surface should be characterized by high biocompatibility, due to the presence of structures based on calcium and phosphates, and have bactericidal properties, due to the presence of zinc and copper ions. Furthermore, the addition of magnesium ions should accelerate the healing of postoperative wounds, which could lead to faster patient recovery.

## 1. Introduction

The phenomenon of luminescence occurring on the surface of metals during the galvanic process was first observed by Sluginov in 1880 [1], and the information was published for the first time by Braun in 1898 [2]. In 1929, Dufford showed that during the electrolysis of metals such as aluminum, zinc, silver, tantalum, tungsten, magnesium, cerium, antimony, and mercury in selected electrolytes, the phenomenon of luminescence was observed [3]. In addition, he noticed that this phenomenon was not observed with lead, iron, copper, nickel, molybdenum, tin, and platinum. Such luminescence phenomenon was developed, *inter alia*, by McNeill, Gruss, Yerokhin, and Nie [4,5]. In addition, Yerokhin at al. proposed a definition of that process, that it is “an electrolytic treatment of anodically polarized metal surfaces carried out above the dielectric breakdown voltage of the growing oxide film”, as well as an explanation of the formation of porous coatings [5,6]. According to this theory, during small voltage polarization, the passive layer is forming, which may be dissolved with the voltage increase. Further increases of voltage result in film repassivation and growth of a new porous oxide film. In the next step of voltage increase, the electric field strength in the oxide film reaches a critical value, after which the film is broken through due to impact or tunneling ionization (here, luminescent sparks are observed). A further increase of voltage results in the following: thermal ionization, larger arc discharges, film fusing and alloying with electrolyte elements, microdischarges penetrating through to the substrate, powerful arcs, destructive effects, and thermal cracking of the coating. It should be also pointed out that on the same plasma electrolytic oxidation (PEO) treated surface, more than one of the described processes may occur concurrently [5]. Over the years, the phenomenon of galvanic luminescence occurring during the anodic polarization of selected metals and electrolytes has been defined as microplasma oxidation, anode spark electrolysis, plasma electrolytic anode treatment, plasma electrolytic oxidation, or micro arc oxidation [7]. Systematic studies proposed coating growth [8,9,10] and discharging [11,12,13,14] behaviors as well as electrolyte influence on the ignition of plasma electrolytic oxidation processes [15]. Meanwhile, Curran and Clyne described the thermophysical properties of plasma electrolytic oxidation [16] and the porosity of PEO coatings [17]. Other authors studied oxygen evolution during PEO [18] and the effects of electrical parameters on that process [19] as well as phase formation in ceramic PEO coatings [20,21] and their corrosion resistance [22]. The PEO process has been performed by researchers under different conditions, i.e., DC [23,24], AC [25,26], and pulse [27,28] currents, which result in different surface morphology [29,30] and chemical composition [31,32] as well as mechanical [33,34] and electrochemical properties [35,36]. For the most frequently treated materials by PEO processes, aluminum [37,38,39,40] and its alloys [40,41,42,43,44], magnesium [45] and its alloys [46,47,48,49,50,51,52,53,54], titanium [55,56,57,58,59,60] and its alloys [61,62,63,64,65,66,67], zirconium [68,69,70,71,72,73,74,75,76,77] and its alloys [78,79], tantalum [80,81,82,83], niobium [84,85,86,87,88], and hafnium [89] should be included. In the present paper, PEO coatings obtained on titanium in novel electrolytes, which have never been used or described in the literature until now, are presented (Table 1). These electrolytes are based on orthophosphoric acid and three different nitrates.

It should also be pointed out that in hydroxyapatite-like structures it is possible to substitute the Ca^2+^ ions for Ca^2+^, Mg^2+^, Cu^2+^, and Zn^2+^, as well as OH^–^ for Cu^+^, which will be used in the fabrication of novel PEO coatings. The porous calcium–phosphate coatings obtained on titanium [117,118,119] and enriched with biocompatible magnesium, which causes faster wound healing [120,121,122,123,124,125], as well as antibacterial zinc [126,127,128,129,130,131,132] and copper [133,134,135,136], may be used as biomaterial, which will be fully accepted by the tissue environment.

However, without results *inter alia* presented in those papers, it is not possible to predict the real possibility of that substitution during plasma treatment in electrolyte in which the ions are present, as well as the thickness and porosity of the PEO coatings. Therefore, in the present paper, the results of x-ray photoelectron spectroscopy (XPS) (10 top nanometers) will be helpful in explaining the oxidation states of those chemical elements as well as chemical composition for all volumes, thicknesses, and pore shapes of obtained coatings by energy dispersive spectroscopy (EDS), XRD, glow discharge optical emission spectroscopy (GDOES), and SEM.

## 2. Materials and Methods

Porous coatings obtained on titanium samples (10 × 10 × 2 mm) by PEO treatment in electrolyte (constant volume of 500 mL for each experiment) containing phosphoric acid (85% *w*/*w*) with the addition of calcium nitrate tetrahydrate Ca(NO_3_)_2_·4H_2_O, magnesium nitrate hexahydrate Mg(NO_3_)_2_·6H_2_O, and copper(II) nitrate trihydrate Cu(NO_3_)_2_·3H_2_O in weight ratios of 1:1:1 (Table 2) at 3 voltages, 500 V (PEO time: 3 min), 575 V (PEO times: 1, 3, 5 min), and 600 V (PEO time: 3 min), using a PWR 1600H power supply (KIKUSUI Electronics Corp., Yokohama, Kanagawa, Japan) were fabricated. For their characterization, the complementary measurement methods SEM, EDS, GDOES, XPS, and XRD [137,138,139,140,141] were used. Descriptions of the setups are presented in Table 3 and are detailed in reference [24].

## 3. Results

**Figure 1:**
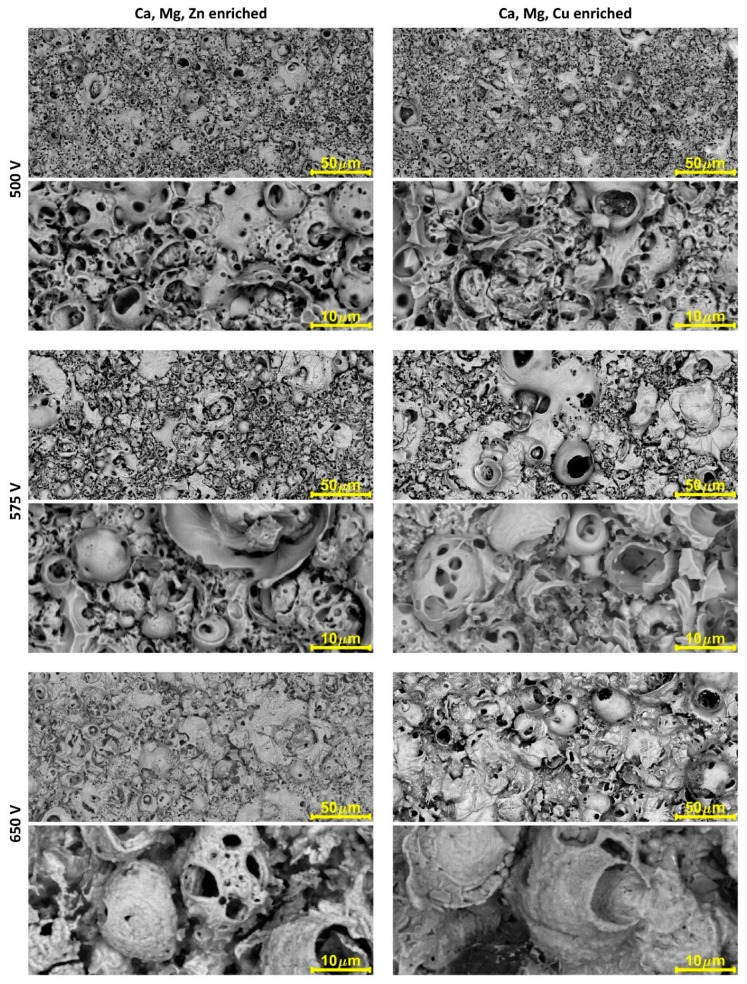
Surface morphologies of surfaces after PEO processing.

Figure 1 shows the surface morphologies of coating surfaces formed on titanium at 500 V, 575 V, and 650 V in two different electrolytes based on phosphoric acid. Two solutions were used: Electrolyte 1, containing H_3_PO_4_ with the addition of calcium nitrate tetrahydrate Ca(NO_3_)_2_·4H_2_O, magnesium nitrate hexahydrate Mg(NO_3_)_2_·6H_2_O, and zinc nitrate hexahydrate Zn(NO_3_)_2_·6H_2_O; and Electrolyte 2, with additions of calcium nitrate tetrahydrate Ca(NO_3_)_2_·4H_2_O, magnesium nitrate hexahydrate Mg(NO_3_)_2_·6H_2_O, and copper(II) nitrate trihydrate Cu(NO_3_)_2_·3H_2_O. It should be pointed out that all the obtained coatings were porous and had a well-developed surface.

**Figure 2:**
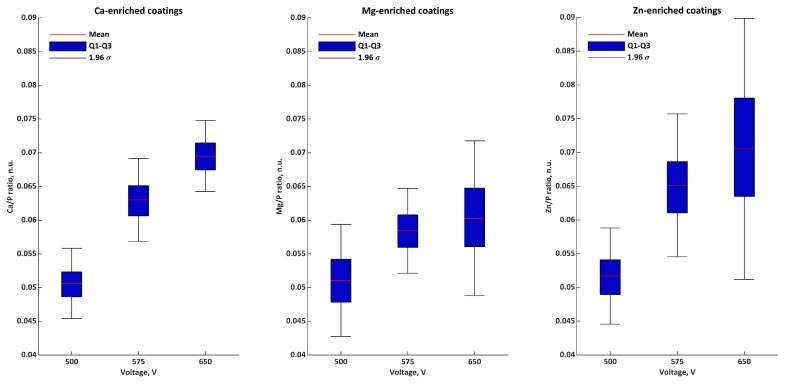
Atomic ratios (EDS) of coatings formed in Electrolyte 1.

In Figure 2 and Table 4, the EDS semiquantitative results for samples obtained in Electrolyte 1 are presented as Ca/P, Mg/P, Zn/P, and M/P ratios. The Ca/P ratios were equal to 0.051 ± 0.003 natural units (n.u.), 0.063 ± 0.003 n.u., and 0.069 ± 0.003 n.u. for 500, 575, and 650 V, respectively. The Mg/P ratios were equal to 0.051 ± 0.004 n.u. (500 V), 0.058 ± 0.003 n.u. (575 V), and 0.060 ± 0.006 n.u. (650 V). The Zn/P ratios for 500, 575, and 650 V were equal to 0.052 ± 0.004 n.u., 0.065 ± 0.005 n.u., and 0.071 ± 0.010 n.u., respectively. The M/P ratios were equal to 0.153 ± 0.008, 0.187 ± 0.006, and 0.200 ± 0.020 for 500, 575, and 650 V, respectively.

**Figure 3:**
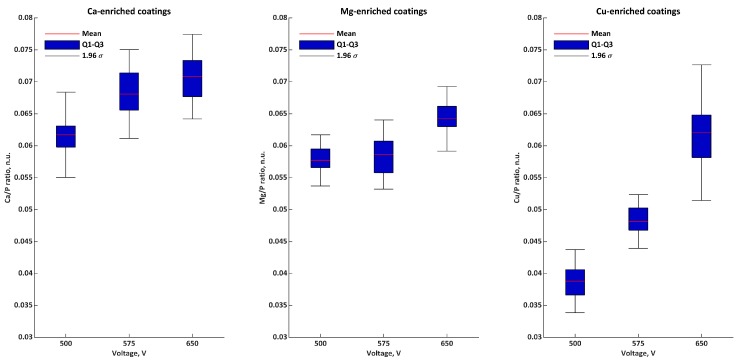
Atomic ratios (EDS) of coatings formed in Electrolyte 2.

Figure 3 and Table 5 present the EDS semiquantitative results for samples obtained in Electrolyte 2 as Ca/P, Mg/P, Cu/P, and M/P. The Ca/P ratios were equal to 0.062 ± 0.003 n.u., 0.068 ± 0.004 n.u., and 0.071 ± 0.003 n.u. for 500, 575, and 650 V, respectively. The Mg/P ratios were equal to 0.058 ± 0.002 n.u., 0.059 ± 0.003 n.u., and 0.064 ± 0.003 n.u. for 500, 575, and 650 V, respectively. The Cu/P ratios for samples obtained at 500, 575, and 650 V were equal to 0.039 ± 0.003 n.u., 0.048 ± 0.002 n.u., and 0.062 ± 0.005 n.u., respectively. The M/P ratios for samples obtained at 500, 575, and 650 V were equal to 0.158 ± 0.006 n.u., 0.175 ± 0.006 n.u., and 0.197 ± 0.004 n.u., respectively.

**Figure 4:**
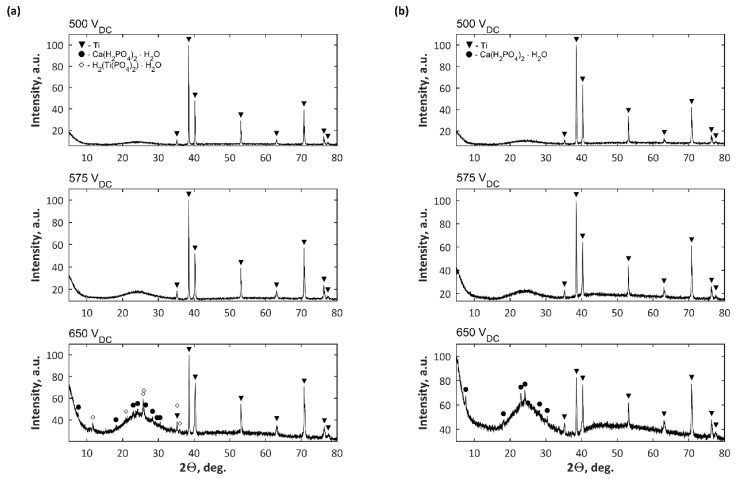
Diffractogram XRD results of PEO coatings obtained in (**a**) Electrolyte 1 and (**b**) Electrolyte 2.

The diffraction data of PEO coatings formed in Electrolytes 1 and 2 at three voltages are presented in Figure 4. For both electrolytes, similar phenomena were observed, i.e., for samples oxidized at 500 and 575 V, only signal from titanium as metal matrix was detected, while for 650 V other crystalline phases, such as Ca(H_2_PO_4_)_2_∙H_2_O and Ti(HPO_4_)_2_∙H_2_O for samples obtained in Electrolyte 1 and Ca(H_2_PO_4_)_2_∙H_2_O for samples obtained in Electrolyte 2, were recorded. It was also found that voltage growth in PEO coatings caused amorphous phase accretion as well.

**Figure 5:**
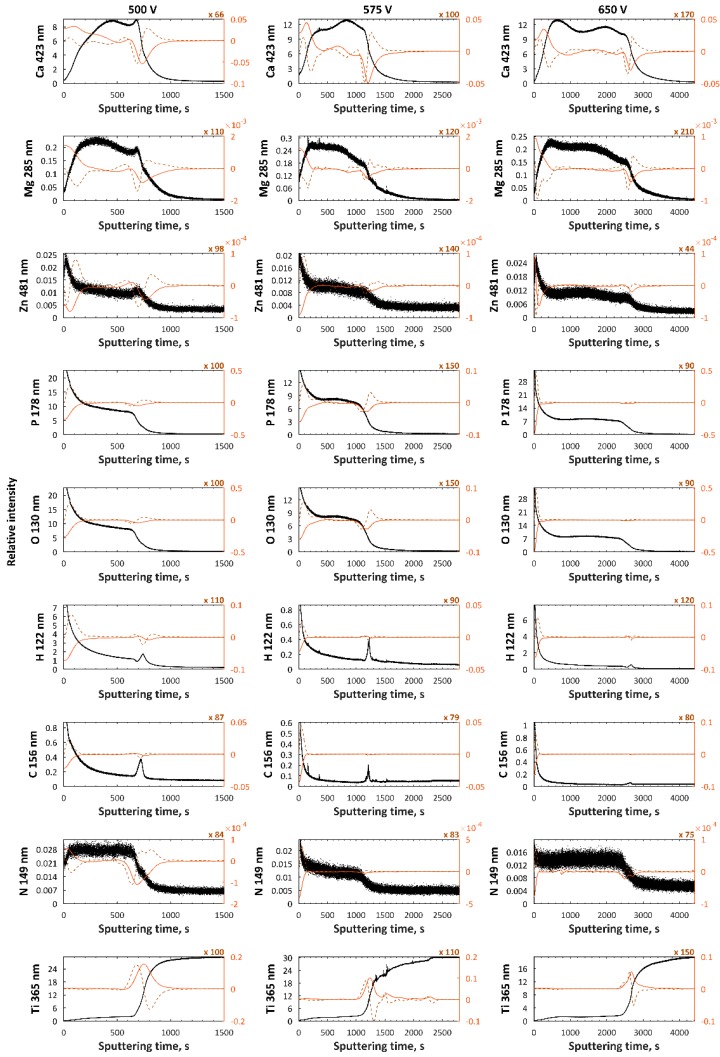
GDEOS signals (black), first derivatives (red continuous line), and second derivatives (brown dashed line) for samples formed in Electrolyte 1.

**Figure 6:**
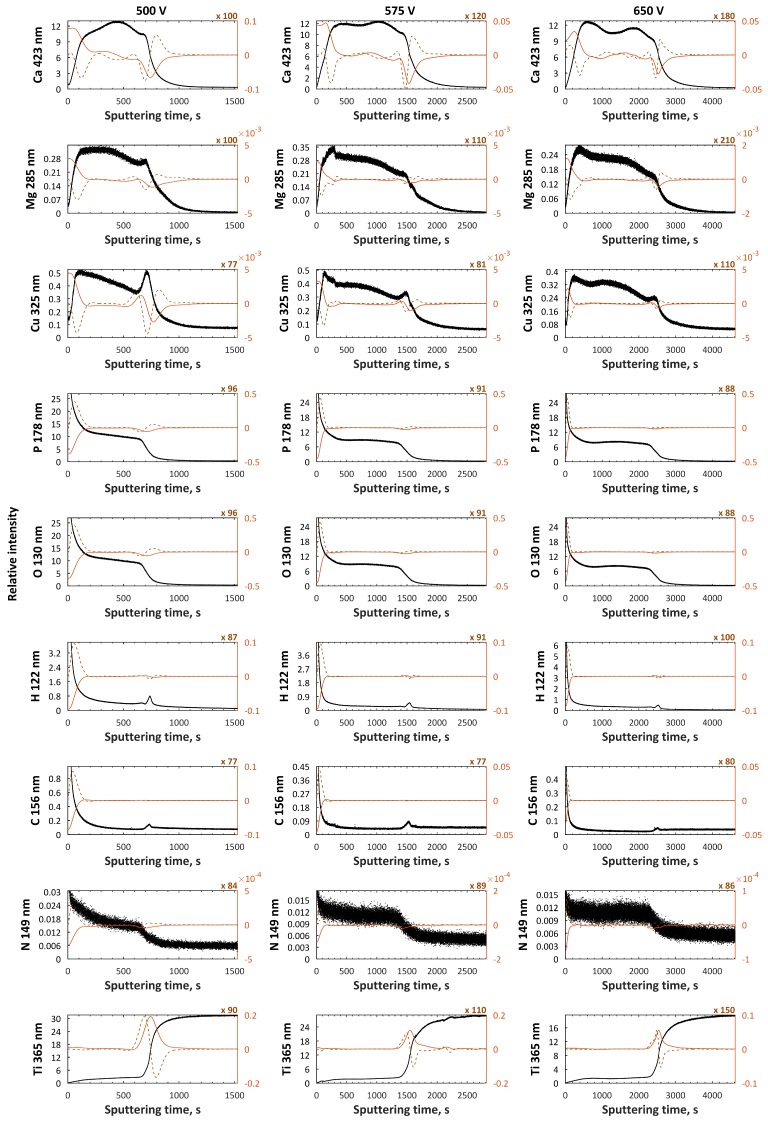
GDEOS signals (black), first derivatives (red continuous line), and second derivatives (brown dashed line) for samples formed in Electrolyte 2.

GDOES data of PEO coatings formed in Electrolyte 1 at 500, 575, and 650 V are presented in Figure 5. The top and porous sublayers, which are enriched in Zn, P, and O and depleted in Ca, Mg, and Ti, have thicknesses of about 200, 300, and 500 s of sputtering time for 500, 575, and 650 V, respectively, while the thickness of the second (semiporous) one, which was enriched in calcium, magnesium, zinc, phosphorus, and oxygen and depleted in titanium, was in the range of 700 s (500 V) up to 2000 s (650 V) of sputtering time. On the other hand, the thicknesses of the third (transition) sublayers, in which a decrease of all signals, except titanium, was observed, increased from 800 s (500 V) up to 2000 s (650 V) of sputtering time. In Figure 6, the GDOES results of PEO coatings formed in Electrolyte 2 at the same three voltages are presented.

**Figure 7:**
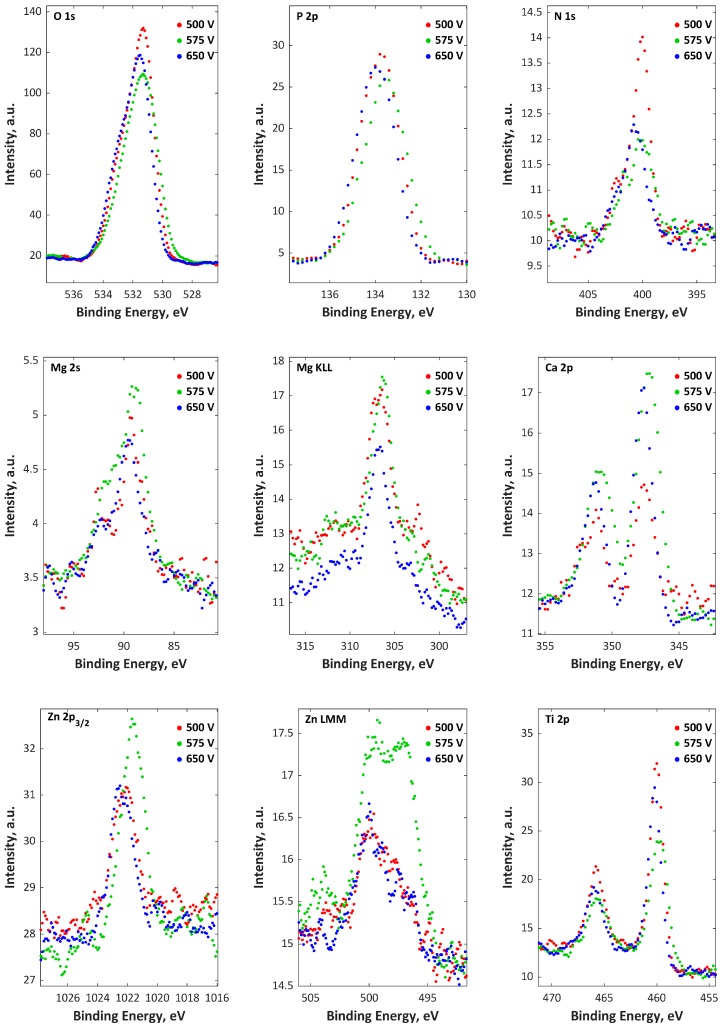
XPS spectra for titanium samples after PEO treatment in Electrolyte 1.

**Figure 8:**
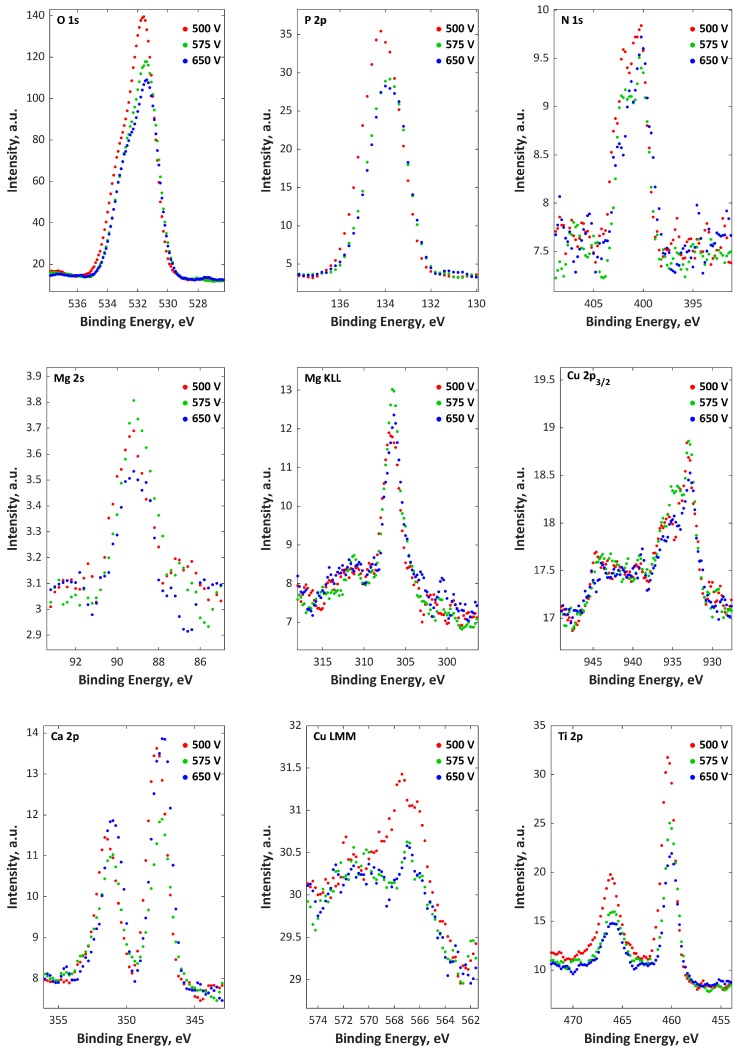
XPS spectra for titanium samples after PEO treatment in Electrolyte 2.

The top and porous sublayers, which are enriched in P and O and depleted in Ca, Mg, Cu, and Ti, have thicknesses related to sputtering times equal to about 100, 300, and 600 s for 500, 575, and 650 V, respectively, while the thickness of the second (semiporous) layer, which is enriched in Ca, Mg, Cu, P, and O and depleted in Ti, is in the range of 600 s (500 V) up to 1900 s (650 V) of sputtering time. Here, the thicknesses of the transition sublayers are in the range from 600 s (500 V) up to 1500 s (650 V) of sputtering time. The part of C, N, and O signals may originate in the first top sublayers from contamination (from air and cleaning compounds). In addition, the H signals maxima, which are always placed in third-transition sublayers, is the end of the coating porosity. It should also be noted that the accretion of voltage caused an increase in coating thickness. In Figure 7 and Figure 8, the XPS spectra of PEO coatings formed in Electrolytes 1 and 2 are presented. Based on the obtained results, it can be concluded that the top external 10 nm layers of the PEO coating consist mainly of phosphorus, oxygen, nitrogen, titanium, calcium, magnesium, and zinc (Electrolyte 1) or copper (Electrolyte 2). The bindings of C with O and N with O can be interpreted as contaminants (cleaning process and adsorbed air). The phosphorus (P 2p) and oxygen (O 1s) spectra were in the range of 133.6–134 eV and 531.3–531.5 eV, respectively, which can be interpreted as the groups PO_4_^3−^, HPO_4_^2−^, H_2_PO_4_^−^, and P_2_O_7_^4−^. The Cu 2p spectra maxima (331.1–932.9 eV and 934.5–935.8 eV) and Auger Cu LMM (566–567.2 eV) suggest the presence of Cu^+^ and Cu^2+^, while Ca^2+^ is proved by the binding energy (BE) in the range of 347.4−347.7 eV. The BE of Zn 2p (1021.9–1022.4 eV) and Zn LMM (497.9–501.5 eV) proves the existence of Zn^2+^, while BE in the range of 89.1–92.8 eV (Mg 2s) and 306.2–306.9 eV (Mg KLL) indicates the existence of Mg^2+^. The BE of titanium Ti 2p_3/_is in the range of 459.9−460.2 eV, which means that titanium is on the fourth oxidation state (Ti^4+^). Based on the quantitative XPS of the top 10 nm of PEO coatings obtained in Electrolytes 1 and 2 at three voltages, two ratios, Ca:Mg:Zn and Ca:Mg:Zn, were found. The Ca:Mg:Zn ratios are equal to 8:32:1 n.u. (500 V), 8:28:1 n.u. (575 V), and 14:45:1 n.u. (650 V), while the Ca:Mg:Zn ratios are equal to 5:5:1 n.u. (500 V), 4:7:1 n.u. (575 V), and 6:5:1 n.u. (650 V). (Ca + Mg + Zn)/P and (Ca + Mg + Cu)/P have their maxima equal to 0.48 n.u. and 0.21 n.u., respectively, at 575 V. The same trend was observed for single Me/P ratios, where M ∈ {Ca, Mg, Zn, Cu}, i.e., the maxima were recorded for PEO coatings obtained at 575 V.

## 4. Discussion

In this paper, the characteristics of new porous coatings fabricated at 500, 575, and 650 V in electrolytes based on H_3_PO_4_ and Mg(NO_3_)_2_·6H_2_O, Ca(NO_3_)_2_·4H_2_O with Cu(NO_3_)_2_·3H_2_O, and Zn(NO_3_)_2_·6H_2_O were presented. Information on the chemical composition of the PEO coatings was obtained by use of the XPS method (for the first 10 nm) and EDS and XRD (for the whole volume of the coatings). Based on EDS results, which were recorded for the whole volume of the coatings, it was found that increased PEO voltage results in an increase of the average metal-to-phosphorus ratios (Ca/P, Mg/P, Zn/P, and Cu/P), while XPS analysis of 10 nm showed that the maxima of those ratios are achieved for the values of the central voltage (575 V), which indicates that the coatings are layered, as proven by GDOES elemental profiles. All the PEO coatings can be divided into three sublayers: (i) external porous layer, enriched in P, O, and Zn (Electrolyte 1) and depleted in Ca, Mg, and Cu (Electrolyte 2) and Ti, but also the most contaminated (CO_2_, C_2_H_5_OH); (ii) semiporous layer, enriched in Ca, Mg, P, O, and Zn (Electrolyte 1) or Cu (Electrolyte 2), and depleted in Ti; (iii) transition layer, in which the titanium signal increases and depletion of all other elements (P, O, Ca, Mg, Zn, and Cu) is detected. On the basis of these XPS data, it was possible to conclude that the extreme surface of the coatings most likely consists of titanium (Ti^4+^), calcium (Ca^2+^), magnesium (Mg^2+^), and oxygen with PO_4_^3−^, HPO_4_^2−^, H_2_PO_4_^−^, and P_2_O_7_^4−^ as well as Zn^2+^ or Cu^+^/Cu^2+^. Furthermore, the XRD analysis suggests that increasing voltage results in amorphization of the coatings, with the detection of crystalline phases such as Ca(H_2_PO_4_)_2_∙H_2_O and/or Ti(HPO_4_)_2_∙H_2_O.

It was also observed that using zinc ions as a bactericidal element instead of the copper ions in PEO coatings obtained on titanium substrate results in a drastic increase of magnesium incorporated into the obtained structure, combined with a slight increase of calcium ions. The results presented in this paper may be used to design biocompatible and bactericidal coatings due to the creation hydroxyapatite-like structures, in which the Ca^2+^ may be replaced by others, i.e., Mg^2+^, Zn^2+^, Cu^2+^, and the hydroxy group (OH^−^) by Cu^+^ ions. It should be pointed out that while magnesium accelerates the healing of postoperative wounds, the structure composed of calcium and phosphorus is bone-like. Therefore, zinc or copper added in controlled quantities would perform antibacterial functions, which, together with magnesium, would allow faster healing of postoperative wounds.

## 5. Conclusions

- It is possible to obtain porous calcium–magnesium–phosphate coatings enriched with copper or zinc.
- The higher the voltage of PEO treatment, the thicker the porous coatings.
- The higher the voltage of PEO treatment, the higher the amount of built-in elements coming from the electrolyte and more amorphous phase in coatings.
- The top 10 nm layer of the studied coatings consist mainly of Ti^4+^, Ca^2+^, Mg^2+^ and PO_4_^3−^, HPO_4_^2−^, H_2_PO_4_.

## Figures and Tables

**Table 1:** Electrolytes and conditions of the plasma electrolytic oxidation (PEO) process.

Electrolytes	Voltage Current Density	Ref.
H_2_O, NaAlO_2_, Na_3_PO_4_·12H_2_O, KOH, NaCl	100–900 mA·cm^−2^ (f = 50 Hz)	[7]
H_2_O, Na_3_PO_4_·12H_2_O, KOH, Na_2_SO_4_, (HOCH_2_)_3_CNH_2_, (NH_4_)_2_HPO_4_, C_2_H_7_NO_2_	70 mA·cm^−2^ (f = 50 Hz)	[23]
H_3_PO_4_, Ca(NO_3_)_2_·4H_2_O, Mg(NO_3_)_2_·6H_2_O, Cu(NO_3_)_2_·3H_2_O, Zn(NO_3_)_2_·6H_2_O	500, 575, 650 V	[24]
H_3_PO_4_, Cu(NO_3_)_2_·3H_2_O	450 V	[32]
H_2_O, Na_2_SiO_3_, (NaPO_3_)_6,_ NaAlO_2_ microparticle	80 mA·cm^−2^ (f = 300 Hz)	[33]
H_3_PO_4,_ Mg(NO_3_)_2_·6H_2_O, Zn(NO_3_)_2_·6H_2_O	500–650 V	[35]
H_2_O, Na_3_PO_4_, FeSO_4_	350 V (f = 100 Hz)	[55]
H_2_O, NaAlO_2_, KOH	400 V (f = 2000 Hz)	[56]
H_2_O, (CH_3_COO)_2_Ca·H_2_O, NaH_2_PO_4_·2H_2_O	300, 390 V (f = 900 Hz)	[58]
H_2_O, Ca(CH_3_COO)_2_,Sr(CH_3_COO)_2_	400, 450 V (f = 100 Hz)	[59]
H_3_PO_4_, Ca(NO_3_)_2_·4H_2_O	500, 575, 650 V	[60]
H_2_O, Na_3_PO_4_, Co(CH_3_COO)_2_	350 V (f = 100 Hz)	[61]
H_2_O, Na_3_PO_4_·12H_2_O, Na_2_B_4_O_7_·10H_2_O, Na_3_WO_4_·2H_2_O	50 mA·cm^−2^	[62]
H_2_O, Na_2_SiO_3_, Na_2_CO_3_, NaOH	12 mA·cm^−2^ (f = 100 Hz)	[63]
H_2_O, C_6_H_18_O_24_P_6_, KOH, EDTA-Na_2_, Ca(CH_3_COO)_2_	20, 50, 80 V	[64]
H_2_O, NaAlO_2_, Na_2_SiO_3_, (NaPO_3_)_6_	550 V	[65]
H_2_O, Na_2_HPO_4_, C_4_H_6_O_4_Ca·H_2_O	+400 V/−80 V (f = 250 Hz)	[66]
H_2_O, C_3_H_9_O_6_P, C_4_H_6_O_4_Ca·H_2_O	+400 V/−80 V (f = 250 Hz)	[66]
H_2_O, Na_2_HPO_4_, C_3_H_7_CaO_6_P·H_2_O	+400 V/−80 V (f = 250 Hz)	[66]
H_2_O, (CH_3_COO)_2_Ca·H_2_O, NaH_2_PO_4_·H_2_O	350–500 V (f = 1000 Hz)	[67]
H_2_O, Ca(CH_3_COO)_2_·H_2_O	300 V (f = 1000 Hz)	[90]
H_2_O, (CH_1_COO)_2_Ca, C_3_H_7_Na_2_O_6_P	250–400 V (f = 100 Hz)	[91]
H_2_O, (CH_3_COO)_2_Ca·H_2_O, C_3_H_7_Na_2_O_6_P·5H_2_O	450 V (f = 100 Hz)	[92]
H_2_O, (CH_3_COO)_2_Ca·H_2_O, C_3_H_7_Na_2_O_6_P·5H_2_O	250–500 V (f = 1000 Hz)	[93,94]
H_2_O, Ca(CH3COO)_2_·H_2_O, CaC_3_H_7_O_6_P	190–600 V (f = 660 Hz)	[95,96]
H_2_O, (CH_3_COO)_2_Ca·H_2_O, C_3_H_7_Na_2_O_6_P·5H_2_O	200–500 V (f = 900 Hz)	[97]
H_2_O, Na4P_2_O_7_·10H_2_O and KOH, NaAlO_2_	0–300 V	[98]
Na_2_B_4_O_7_·10H_2_O, (CH_3_COO)_2_Mn·4H_2_O	450–500 V	[99]
H_2_O, (CH_3_COO)_2_Ca·H_2_O	230 V	[100]
H_2_O, (CH_3_COO)_2_Ca·H_2_O, NaH_2_PO_4_·2H_2_O	260–420 V	[101]
H_2_O, CaHPO_4_, Ca(H_2_PO_4_)_2_, Na_6_P_6_O_18_, Ca(CH_3_COO)_2_	20, 100 mA·cm^−2^	[102]
H_2_O, KOH	290 V (f = 100–200 Hz)	[103]
H_2_O, KOH	350 V (f = 1000 Hz)	[104]
H_2_O, (NaPO_3_)_6_, NaF, NaAlO_2_	150–200 V	[105]
H_2_O, K_2_Al_2_O_4_, Na_3_PO_4_, NaOH	400 V	[106]
H_2_O, CaCl_2_ and KH_2_PO_4_	320–340 V	[107]
H_2_O, H_2_SO_4_ and Ti_2_(SO_4_)_3_	1100 V	[108]
H_2_O, Na_2_(EDTA), CaO, Ca(H_2_PO_4_)_2_, Na_2_SiO_3_·H_2_O	350 V (f = 200 Hz)	[109]
H_2_O, Na_2_SiO_3_, NaOH	280 V	[110]
H_2_O, CaO, Na_6_P_6_O_18_, Na_2_H_2_EDTA⋅5.5H_2_O, KOH	AC 0.5–2 mA·cm^−^^2^	[111]
_2_O, (NaPO_3_)_6_, NaF, NaAlO_2_	60 mA·cm^−^^2^ (f = 100, 600 Hz)	[112]
H_2_O, Na_3_PO_4_, FeSO_4_, Co(CH_3_COO)_2_, Ni(CH_3_COO)_2_, K_2_ZrF_6_	350 V (f = 100 Hz)	[113]
H_2_O, Ca(CH_3_COO)_2_·H_2_O, C_3_H_7_Na_2_O_6_P	150 V	[114]
H_2_O, Na_2_SiO_3_·9H_2_O, Na_3_PO_4_·12H_2_O, Na_2_SiO_3_·9H_2_O, Na_3_PO_4_·12H_2_O	80 mA·cm^−^^2^ (f = 150 Hz)	[115]
H_2_O, Na_3_PO_4_·12H_2_O, α-Al_2_O_3_ nanoparticles	20 mA·cm^−^^2^	[116]

**Table 2:** Experimental plan and code sample names.

Sample Name	Voltage	Electrolyte Type	Electrolyte Composition	
			Salts	Salt Concentrations (g/L)
Ti_CaMgZn_500V	500 V	Electrolyte 1	Ca(NO_3_)_2_·4H_2_O and Mg(NO_3_)_2_·6H_2_O & Zn(NO_3_)_2_·6H_2_O	166.7 + 166.7 + 166.7
Ti_CaMgZn_575V	575 V			
Ti_CaMgZn_650V	650 V			
Ti_CaMgCu_500V	500 V	Electrolyte 2	Ca(NO_3_)_2_·4H_2_O and Mg(NO_3_)_2_·6H_2_O & Cu(NO_3_)_2_·3H_2_O	166.7 + 166.7 + 166.7
Ti_CaMgCu_575V	575 V			
Ti_CaMgCu_650V	650 V			

**Table 3:** Setups of SEM, energy dispersive spectroscopy (EDS), x-ray photoelectron spectroscopy (XPS), glow discharge optical emission spectroscopy (GDEOS), and XRD equipment.

Technique	Equipment	Manufacturer
SEM	Quanta 650 FEI	Field Electron and Iron Company, Hillsboro, OR, USA
EDS	Noran System Six	EDS, Silicon Drift Detectors: Keith Thompson, Thermo Fisher Scientific, Madison, WI, USA
XPS	SCIENCE SES 2002	Scienta AB, Scienta Omicron, Uppsala, Sweden
GDOES	GD Profiler 2	HORIBA Scientific, Palaiseau, France
XRD	Bruker-AXS D8 Advance	Bruker Corporation, Billerica, MA, USA

**Table 4:** Statistical description of EDS of coatings formed in Electrolyte 1. n.u., no units.

Ratios	Voltage	$\bar{x}$	σ	Q_1_	Q_2_	Q_3_
Ca/P n.u.	500 V	0.051	0.003	0.050	0.052	0.052
	575 V	0.063	0.003	0.062	0.064	0.065
	650 V	0.069	0.003	0.068	0.071	0.071
Mg/P n.u.	500 V	0.051	0.004	0.049	0.051	0.053
	575 V	0.058	0.003	0.057	0.060	0.060
	650 V	0.060	0.006	0.057	0.063	0.063
Zn/P n.u.	500 V	0.052	0.004	0.050	0.053	0.054
	575 V	0.065	0.005	0.063	0.068	0.068
	650 V	0.071	0.010	0.065	0.075	0.075
M/P n.u.	500 V	0.153	0.008	0.149	0.151	0.157
	575 V	0.187	0.006	0.184	0.188	0.190
	650 V	0.200	0.015	0.192	0.195	0.206

**Table 5:** Statistical description of EDS of coatings formed in Electrolyte 2. n.u., no units.

Ratios	Voltage	$\bar{x}$	σ	Q_1_	Q_2_	Q_3_
Ca/P n.u.	500 V	0.062	0.003	0.060	0.061	0.062
	575 V	0.068	0.004	0.066	0.068	0.071
	650 V	0.071	0.003	0.068	0.072	0.073
Mg/P n.u.	500 V	0.058	0.002	0.057	0.057	0.059
	575 V	0.059	0.003	0.056	0.060	0.061
	650 V	0.064	0.003	0.064	0.064	0.066
Cu/P n.u.	500 V	0.039	0.003	0.037	0.040	0.040
	575 V	0.048	0.002	0.047	0.048	0.050
	650 V	0.062	0.005	0.059	0.061	0.063
M/P n.u.	500 V	0.158	0.006	0.156	0.156	0.159
	575 V	0.175	0.006	0.172	0.176	0.177
	650 V	0.197	0.004	0.195	0.196	0.197

## Nomenclature

PEO	Plasma electrolytic oxidation
MAO	Micro arc oxidation
SEM	Scanning electron microscopy
EDS	Energy dispersive spectroscopy
GDOES	Glow discharge optical emission spectroscopy
XPS	X-ray photoelectron spectroscopy
XRD	X-ray powder diffraction
$\bar{x}$	Mean
σ	Standard deviation
Q_1_	First quartile
Q_2_	Second quartile (median)
Q_3_	Third quartile
M	Metal (here M = Ca + Mg + Zn or M = Ca+ Mg + Cu)
BE	Binding energy
f	Frequency
DC	Direct current
AC	Alternating current
n.u.	no unit
